# Swine Influenza Virus Introduction in Pig Farms: A Semi-Quantitative Risk Assessment in Northern Italy

**DOI:** 10.3390/ani16040544

**Published:** 2026-02-10

**Authors:** Alessia Rusinà, Alessandro Bellato, Annalisa Scollo, Alessandro Mannelli, Laura Tomassone

**Affiliations:** Department of Veterinary Sciences, University of Turin, 10095 Grugliasco, Italy; alessia.rusina@unito.it (A.R.); alessandro.bellato@unito.it (A.B.); annalisa.scollo@unito.it (A.S.); alessandro.mannelli@unito.it (A.M.)

**Keywords:** swine influenza, risk assessment, biosecurity

## Abstract

Swine influenza is a viral zoonotic respiratory disease of pigs threatening both animal and human health due to its pandemic potential. We conducted a semi-quantitative risk assessment to classify 22 commercial pig farms in northern Italy in terms of the likelihood of introduction of influenza, evaluating their biosecurity systems and geographical locations. We used a modified Failure Mode and Effect Analysis (FMEA) to calculate Risk Priority Codes (RPCs), indicating increasing risk levels (from 1 to 5) for six biosecurity criteria: (A) personnel, (B) animal management, (C) shelter management, (D) animal transport, (E) materials management, and (F) buildings and access control. Then, an overall risk index, including the geographical risk, was calculated to obtain the final risk ranking of the farms. All farms obtained the highest risk level (RPC = 5) for criterion F (buildings and access control), mostly due to poor control of visitor access. Fifteen farms scored a medium risk level (RPCs = 3) for criterion related to personnel due to some weaknesses in the use of personal protective equipment, although 90% of personnel had recent biosecurity training. In an endemic context, risk assessment helps identify high-risk farms, supporting surveillance for early detection of new influenza strains.

## Simple Summary

Influenza viruses are a major health priority worldwide. Pigs can be infected with both human and avian strains, increasing the risk of emergence of new strains with pandemic potential. Therefore, risk-based surveillance for the early detection of new viruses is needed. In our study, we classified 22 commercial pig farms in northern Italy in terms of likelihood of influenza virus introduction, based on their biosecurity system and geographical location. We used a semi-quantitative risk assessment method to calculate risk priority codes, indicating increasing levels of risk for biosecurity criteria for the evaluated farms. Then, an overall risk index was calculated including a geographical risk indicator. The highest risk for all the farms regarded biosecurity related to buildings and access control, due to poor visitor control. Medium risk was obtained in fifteen farms for biosecurity related to personnel, with most of the farms compliant with training on biosecurity, but with some critical points related to the use of personal protective equipment. The method allowed the identification of farms at higher risk, which is crucial to implement risk-based surveillance and to apply strategies aimed at risk mitigation.

## 1. Introduction

Swine influenza (swIA) is a highly contagious and zoonotic disease of pigs caused by type A influenza viruses (IAV) belonging to the Orthomyxoviridae family [1], characterized by high morbidity in pigs reaching up to 100%. The mortality is usually less than 1% [2], although it can increase up to 10–15% when infection occurs in naïve pigs [3]. Pigs can shed virus from 24 h after infection, for up to 7–10 days [4]. The clinical signs of an acute outbreak include lethargy, high fever, anorexia, and coughing. Their onset depends on various factors, including the age and immune status of the host, climatic conditions, and housing; the disease often remains subclinical [2]. IAV are single-stranded RNA viruses with a segmented genome comprising eight segments encoding different proteins [5]. Among the species which can harbor IAV, pigs are of particular concern, as they can be simultaneously infected with avian and human strains [6]. This favors antigenic drift and reassortment, increasing the chance of creating new influenza strains with pandemic potential [7]. This happened with the most recent human IAV 2009 pandemic, which was at least partially derived from swine viruses [8]. The predominant strains circulating in pigs in Europe today are H1N1, H1N2, and H3N2 and derived from a genetic reassortment of human, avian, and pig strains [6]. Swine influenza is endemic in Europe. A surveillance study carried out between 2015 and 2018 estimated a herd level prevalence of 56.6% [6].

The zoonotic potential of swine influenza represents a threat to both animal and public health [9] and is a global concern. Consistently, swine influenza is one of the ten priority zoonotic diseases listed by the European Food Safety Authority (EFSA) for the establishment of a coordinated surveillance system under the One Health approach [10]. Moreover, zoonotic influenza viruses are considered among the prioritized zoonotic diseases [11]. Given the number, density, and movement of animals, intensive farming systems are of high relevance for the potential zoonotic transmission of influenza viruses [12].

Although the benefits of surveillance to public health can be easily recognized, a surveillance program for swIAV is less appealing to farmers, due to the perception that influenza has little impact on pig performance and for the concerns about sanitary restrictions [13]. Moreover, when carried out, surveillance is usually based on sampling of pigs showing clinical signs or included in routine testing for other diseases, thus resulting in a low likelihood of viral detection in endemic herds with low prevalence [13,14]. As a result, the virus circulation may remain unnoticed, allowing multiple strains to co-exist within the same herd or animal [15,16,17] and persist in farms [18,19]. Considering the characteristics of the disease and its mode of transmission, as well as the epidemiological situation in Europe, a risk-based surveillance approach could enhance the detection of new circulating strains in herds. This approach would focus surveillance efforts on farms at a higher risk of viral introduction, rather than relying on clinical signs of the disease.

Risk-based surveillance strategies often rely on risk assessment, a transparent process for estimating the probability and consequences of the introduction of a pathogen in animal populations used to direct decision-making and mitigation measures. The evaluation of biosecurity to assess the risk of introduction of a pathogen in a farm is key in animal health risk assessment. Written protocols to collect data to assess biosecurity are commonly used, especially in the pig farming sector, where the output is usually a score based on the measures applied on farm [20]. Most widely used biosecurity assessment methods do not target a specific pathogen, such as the Biocheck.Ugent [21], or combine biosecurity with other aspects like antimicrobial resistance or pig welfare, as in the Italian system Classyfarm [22]. Disease-specific biosecurity assessments for pigs have been implemented for few diseases, including African Swine Fever (ASF) and porcine reproductive and respiratory syndrome [20]. However, to the authors’ knowledge there is no biosecurity assessment that targets the risk of IAV in pig farms and takes into consideration the peculiar characteristics of this virus and its transmission dynamics.

The primary route of swIAV transmission in pigs is direct contact with infected animals, most commonly when new animals are introduced to a farm, where they come into contact with a susceptible population [23]. Additionally, the ongoing birth of susceptible piglets supports the persistence of the virus within herds [23]. Airborne transmission has also been described [24], along with transmission through contaminated fomites [25]. The potential for virus introduction through humans and birds must also be considered. Consequently, both managerial factors (e.g., husbandry practices) and geographical factors (e.g., farm’s location in relation to other pig or poultry farms or the presence of wild birds) may influence swIA risk.

In this study, we applied a semi-quantitative risk assessment method to rank a sample of pig farms in northern Italy according to their risk of introduction and circulation of swIAV, based on the evaluation of their biosecurity systems. Northern Italy is characterized by intensive livestock production, which involves a high volume of vehicle, animal, and human movements. The assessment integrated biosecurity data with spatial data to account for geographical risk factors, such as the suitability of the area to high pathogenicity avian influenza (HPAI) virus circulation in both wild and domestic birds. This allowed us to identify critical points in biosecurity that might favor the introduction of influenza viruses. In the context of risk assessment, a semi-quantitative approach is useful for guiding interventions, because it uses a scoring system to evaluate risk and classify elements according to their level of risk. Prioritizing farms according to the risk may support targeted allocation of resources and improve the efficiency of surveillance and monitoring systems.

## 2. Materials and Methods

### 2.1. Study Area and Farm Selection

The study was conducted on pig farms located in the northern Italian regions of Piedmont, Lombardy, and Emilia–Romagna. Together, these regions account for over 75% of the national pig population and report the highest animal density, with a total of 4094 commercial pig farms rearing almost 6 million animals [26]. Lombardy has the highest average pig density, with 157 pigs per square kilometer, compared to the national average of 26 pigs per square kilometer. This area is characterized by the intensive farming of different animal species relevant for the transmission of influenza viruses, such as poultry, with almost 40% of the Italian poultry population reared in those three regions [26].

The farms included in this study were selected based on the farmers’ availability, as identified by three pig veterinary practitioners. The farmers were provided with explanations on the assessment, and informed consent was obtained.

High numbers and density of animals are recognized risk factors for swIAV; therefore, there was a specific interest in focusing on commercial intensive farms in highly populated areas.

### 2.2. Checklist and Data Collection

To collect data on farms’ biosecurity, a checklist previously developed by Scollo et al. [27] for African swine fever was modified and adapted to swine influenza. The checklist was adapted following an extensive literature review and existing checklists for pig biosecurity and considers the risk factors, pathways, and conditions that may favor the introduction and circulation of swIAV into a farm.

The checklist is composed of six criteria (Table 1), 27 sub-criteria, and 132 items. Each criterion represents a risk pathway, i.e., one of the possible routes of swIAV introduction into farms. A criterion can thus be seen as a sector of the biosecurity management system that encompasses several aspects to be considered for virus introduction. Each criterion consists of sub-criteria, with each sub-criterion grouping several items. Each item represents a single question, most of the questions requiring a dichotomous answer (‘Yes’/‘No’), where a ‘Yes’ answer means compliance with a certain biosecurity measure. The checklist allows for the assessment of different production types and enables users to select ‘Not applicable’ for biosecurity measures that cannot be applied to certain production types. For example, ‘Quarantine of at least six weeks is carried out’ could be considered not applicable for weaning and fattening sites, where other strategies such as ‘all-in–all-out’ are usually adopted.

An experienced assessor collected data through direct observations in the farms and face-to-face interviews with farm workers in the period June 2024–February 2025. The assessor always acted according to good biosecurity practices. Depending on the farm type, it generally took two hours to complete the checklist.

**Figure 1:**
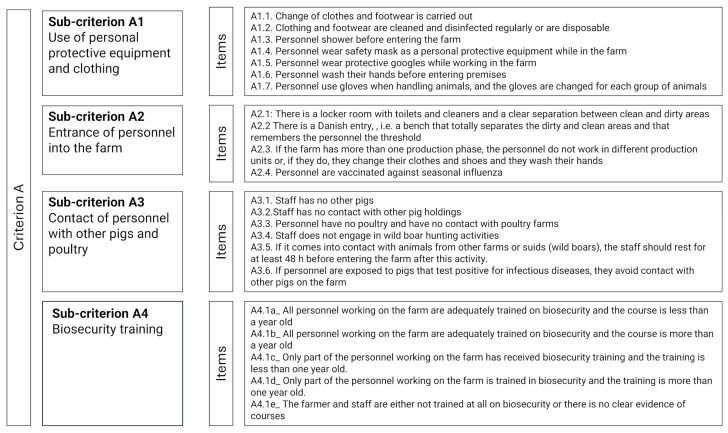
Extract of the swIA checklist used for data collection on farms. Criterion A comprises four sub-criteria (A1, A2, A3 and A4). Each sub-criterion comprises several items (A1.1, A1.2, …, A4.1e).

For clarity, an extract of the checklist is presented in Figure 1. The checklist comprising all the items evaluated on farms is provided in the Supplementary Materials, Table S1.

### 2.3. Farm Categorization Method

#### 2.3.1. Importance Score

As there is little literature available on the efficacy of biosecurity measures for swIAV, a survey was launched through the EU survey platform [28] to obtain expert opinions on the importance of such measures. In the survey, experts were asked to provide an importance score from 1 (very low importance) to 5 (very high importance) for each of the 27 sub-criteria of the checklist. They were also asked to identify measures that could compromise a farm’s entire biosecurity system if not implemented, hereafter referred to as ‘critical items’.

To select experts, a purposive sampling was adopted [29]. Fifteen professionals from different European countries, belonging to the European Network on Swine Influenza (CA21132) and related pig expert networks, answered the survey. They had backgrounds in swine biosecurity, pathology and/or epidemiology, and all had at least 10 years of professional experience.

Importance scores retrieved from the survey were combined to classify the sub-criteria within each criterion from the most to the least important using a modified Borda method, as previously described [27]:

(1) $$I_{b\left(x\right)}=\sum_{\left\{i=1\right\}}^{m}I_{i\left(x\right)}$$

where *I_b_* is the Borda score of a sub-criterion *x*, calculated as the sum of the importance scores assigned by expert *i*, for *i* = 1, 2, … *m* (*m* = 15). Within each criterion, the sub-criterion with the highest *I_b_* is considered the most important and was assigned a score of 5. The scores of the other sub-criteria were subsequently calculated in decreasing order according to their *I_b_* scores.

#### 2.3.2. Non-Compliance Score for Sub-Criteria

Each sub-criterion was assigned a score from 1 (high compliance) to 5 (low compliance), based on compliance with the relevant biosecurity measures of the checklist. The score increased as the proportion of non-compliant measures within that sub-criterion increased, as detailed in Table 2. Non-compliance with any critical item identified by experts resulted in the assignment of the maximum non-compliance score (5) to the corresponding sub-criterion.

In a few cases, sub-criteria were composed of mutually exclusive response options, each corresponding to a different level of non-compliance (e.g., sub-criterion A4, Figure 1). In these cases, a score from 1 to 5 was directly assigned based on the selected response, reflecting the increasing level of non-compliance.

#### 2.3.3. Calculation of Risk Priority Codes

A modified Failure Mode and Effects Analysis (FMEA) method [27] was applied to score the criteria. This semi-quantitative method enables the calculation of Risk Priority Codes (RPCs), which reflect increasing levels of risk associated with specific elements within a system. The importance scores and the non-compliance scores attributed to the sub-criteria were used to compute an RPC for each criterion. The RPC ranges from 1 to 5, indicating increasing levels of risk.

Analyses were performed using R, version 4.2.0 [30]. The RPC was calculated using Equation (2).

(2) RPC(ai)=Maxj{Min[(Igj),gj(ai)]}

where *RPC*(*ai*) is the Risk Priority Code for the criterion *ai* (with *i* = A, B, …, F); *gj*(*ai*) is the non-compliance score of each sub-criterion *j* (with *j* = A1, A2, …, *n*) included in the criterion *ai* (calculated following Table 2); and *Igj* is the importance score of the sub-criterion *j*, included in criterion *ai*, calculated using the modified Borda method.

Equation (2) corresponds to the second analysis model described by Franceschini and Galetto [31]. The RPC of a criterion is the maximum value between the minimum values of the sub-criteria’s importance and the scores assigned to those sub-criteria. This method enables the weighting of non-compliance with the most important biosecurity measures for preventing swIA virus entry, as determined by experts. The objective is to assign a high RPC to farms with the highest non-compliance score (i.e., poor biosecurity) on the most important sub-criteria.

#### 2.3.4. Geographical Risk Factors

To account for the risks posed by poultry and wild birds, the farm’s geographical location was considered in relation to ecological niche suitability for H5Nx viruses in both domestic poultry and wild birds, as provided by Dupas et al. [32] on the MOOD (MOnitoring Outbreaks for Disease surveillance in a data science context) platform [33].

Maps of domestic and wild birds H5Nx viruses’ suitability were downloaded from the MOOD platform and imported onto QGIS version 3.16.2 [34]. Using known coordinates of geographical waypoints, the pictures were reprojected as raster layers into WGS 84 Datum reference system. This procedure was intended to ensure spatial alignment between suitability surfaces and farm locations, rather than to recover absolute metric properties of the original rasters. The three-band color values—Red, Green, Blue (RGB)—were extracted using the ‘rasterio’ package in Python (version 1.4.3) [35]. RGB values were converted to scalar values using a weighted interpolation based on their proximity to a set of reference color samples, derived from the map legend. For each pixel, the Euclidean distance in RGB space was computed between its color and all reference samples. The two nearest reference colors, with scalar values, and corresponding distances *d*_1_ and *d*_2_, were then used to compute an interpolated scalar value for that pixel as

(3) $$s\;=\;v_{1}\times \;\left(\frac{d_{2}}{d_{1}\;+\;d_{2}}\right)\;+\;v_{2}\;\times \;\left(\frac{d_{1}}{d_{1}\;+\;d_{2}}\right)$$

This results in a smooth interpolation in the RGB space, mapping continuous colors to their corresponding scalar values. Using the QGIS plugin sample raster value, we extracted the values from the two raster images for each pig farm censused in Italy. Suitability values were used exclusively for relative ranking of farms within each raster layer. Italian pig farms were ranked separately according to their suitability values for H5Nx viruses in domestic and wild birds, resulting in two indicators. Quintiles were calculated, and a score from 1 (low suitability) to 5 (high suitability) for both indicators was assigned to each pig farm sampled in our study. Finally, the maximum between the two scores was selected as the RPC to represent the geographical risk.

#### 2.3.5. Overall Classification of Farms

Each farm was assessed through seven risk indicators. Six were related to biosecurity (i.e., criteria assessed through the on-farm checklist) and one was related to the geographical risk, by considering the suitability of the area for HPAI.

To obtain an aggregated risk index, for each farm we calculated the counts of decreasing values for each of the seven risk indicators, from counts of 5 to counts of 1. A risk ranking was then assigned to each farm by sorting them in descending order. The highest risk was attributed to those farms characterized by the highest frequency of RPCs = 5. Among farms with the same number of 5 s, the one with the highest number of 4s was classified as posing the highest risk. Then, the counts of 3 s and 2 s were considered. This process yielded an overall ranking of farms, from the highest (rank = 1) to the lowest risk of IAV introduction.

## 3. Results

### 3.1. Survey Results on Importance of Biosecurity Measures

Importance scores of the sub-criteria, according to their *I_b_*, are reported in Table 3. Overall, the most important sub-criterion (highest *I_b_*) was A4, ‘Biosecurity training of the personnel’. Table 4 shows the critical items; the cut-off for inclusion in the list was set at a minimum of 13 experts selecting the item.

### 3.2. Farm Assessment

The checklist was completed for 22 commercial pig farms. The number of animals housed on the farms ranged from 750 to 6000 (not including the number of suckling piglets at the farrowing sites). Of these farms, eight were located in Piedmont region, nine in Lombardy, and five in the Emilia–Romagna region, and they presented different production types (farrow-to-finish, farrowing, weaning, fattening sites, and mixed type).

A total of 2582 records of compliance information (‘yes’ or ‘no’) were obtained for single biosecurity measures, corresponding to the 132 items, while 322 records were classified as ‘not applicable’. A total of 24 records were missing and could not be recovered and were therefore considered as ‘not applicable’.

**Figure 2:**
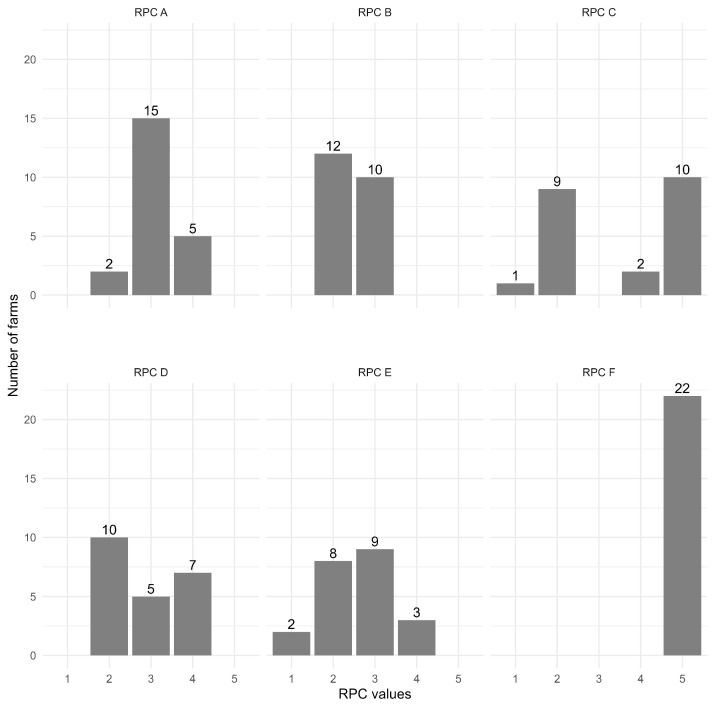
Distribution of risk priority codes for six biosecurity criteria (A to F), across 22 evaluated farms.

The risk priority codes calculated for the 22 assessed farms are summarized in Figure 2, and the main descriptive statistics are described below. Details of the non-compliance scores are reported in the Supplementary Materials, Table S2.

#### 3.2.1. Criterion A: Personnel

Fifteen farms received an RPC score of 3, indicating a medium risk in relation to the implementation of biosecurity measures for personnel (Figure 2). The non-compliance scores for the sub-criteria used to calculate the RPC for criterion A are summarized in Table S2. A high frequency of a non-compliance score of 3 was observed for sub-criterion A1 (Use of personal protective equipment and clothing), which is explained by the fact that personnel did not shower before entering in more than half of the farms. Additionally, 86.4% did not wear safety masks, and 100% did not use protective goggles. In all the farms, the personnel changed footwear and clothes (A1_1), which was considered a critical item. For sub-criterion A2 (Entrance of personnel into the farm), we recorded the highest level of non-compliance for item A2_4, with more than 75% of farms reporting that their personnel were not vaccinated against influenza.

Regarding sub-criterion A3 (Contact of personnel with other pigs and poultry), in more than half of the farms, personnel had contact with other pig holdings, and in 22.7% personnel had contact with poultry. No farms reported personnel engaging in wild boar hunting.

Sub-criterion A4 (Biosecurity training) showed the lowest non-compliance, with 20 out of 22 farms reporting that all staff had received biosecurity training within the last year.

#### 3.2.2. Criterion B: Animal Management

For Criterion B, related to animal management, all farms received RPCs indicating low–medium (RPC = 2) or medium risk (RPC = 3) (Figure 2). B3 (Number of farms of origin of the introduced pigs), B4 (Number of animal introductions per year), and B5 (Management of animals with an impaired growth) are sub-criteria with mutually exclusive answers, indicating an increasing level of non-compliance, from 1 to 5 (=higher level of non-compliance). In criterion B, sub-criterion B4 showed the highest non-compliance scores (median = 3) (Table S2), with almost half of the farms introducing animals 3–5 times a year. In contrast, 77.2% of farms introduced new pigs from at most two farms, providing a low non-compliance score for sub-criterion B3. Regarding sub-criterion B1 (Animals health status/identification/recording of movements and productions), in most of the farms, animals were not vaccinated against swIAV, and no animal was tested for swIAV prior to its introduction in the farm. In contrast, all farms registered animal entering and leaving the farm, with animals correctly identified and documented vaccination plans against other diseases.

#### 3.2.3. Criterion C: Shelter Management

For Criterion C (Shelter management), ten farms obtained the highest risk level (RPC = 5) (Figure 2). Four critical items were identified for this criterion by experts. The highest risk scores resulted from non-compliance to critical items C1_8, and C2_1 related to all-in–all-out system management. In detail, all-in–all-out in the whole farm or a suitable period of quarantine was not applied in 45.5% of farms. On the contrary, the remaining 12 farms received the highest compliance score (1) to sub-criterion C2 (Internal animal flow and cleaning and disinfection procedures).

All farms were compliant to the other two critical items C1_3 (Presence of a physically separate premise dedicated to quarantine (if applicable) and C2_5 (Presence of a cleaning and disinfection protocol).

For sub-criterion C5, related to measures for the sick pen, most of the farms received a non-compliance score of 3. Overall, 21 farms had a space designated as a sick pen for animals, which was physically separated from the other pens in 80% of farms, with no direct contact between healthy and sick animals. However, only in three farms the sick pen had a separated ventilation system from the rest of the facilities.

#### 3.2.4. Criterion D: Animal Vehicles

The highest RPC score obtained for this criterion is 4 (medium–high risk), which was assigned to seven farms. Ten farms obtained an RPC = 2, indicating medium–low risk (Figure 2).

No critical items were identified from the experts for this criterion. Several farms (n = 13) were compliant to all the biosecurity measures of sub-criterion D3 related to carcass management (non-compliance score = 1), and of sub-criterion D4 (Equipment and tools for loading/unloading live animals) (11 farms). In detail, in more than 70% of the farms, the carcass removal truck does not enter the farm, with most of the farms having the carcass storage outside of the clean area. In 80% of farms, there was no contact between the carcass storage and the carcass truck when emptying. Regarding the loading/unloading of animals, in more than 90% of farms, transporters do not enter any clean area. However, in almost 40% of farms, the clothing provided to transporters is not company issued nor freshly laundered.

#### 3.2.5. Criterion E: Material Management: Feed and Fodder, Slurry, Other Vehicles

RPCs from Criterion E were not homogeneous; most of the farms obtained a score equal to 2 or 3 (low–medium and medium risk). No one obtained the highest risk level (RPC = 5) (Figure 2).

Fifteen farms had the highest compliance for sub-criterion E1 (Procedures for loading/unloading of feed and materials). In 72.7% of farms, the unloading of materials, such as feed or bedding, took place outside the clean area. Regarding sub-criterion E2 (Feed and materials storage), in all farms, feed storage is protected from wild and peridomestic animals. Twenty out of 21 farms did not exchange materials, equipment, or feed with other farms.

Farms received higher scores (higher non-compliance) for sub-criterion E3 (Slurry management). Indeed, most farms do not prevent access to the slurry tank by wild animals, and in more than 20% of the farms, the slurry tank is located within the clean area. Almost all the farms do not allow slurry from other farms to be spread in adjacent fields.

#### 3.2.6. Criterion F: Buildings and Access Control

All farms received the highest RPC score (5) for criterion F (Figure 2). This is due to the fact that none of the farms complied with measure F4_5, which was considered a critical item, i.e., no farm prevented visitors showing clinical signs of influenza from entering the premises.

Non-compliance with a critical item automatically raises the non-compliance score of the corresponding sub-criterion to the maximum value (5). In this case, not only is F4_5 a critical item, but the importance score of sub-criterion F4 (Visitors) is also 5. As a result, applying Equation (2) led to an RPC score of 5 for all farms.

Moreover, in 86.4% of farms, visitors did not wear masks when entering. Other issues were also observed in the sub-criterion related to visitor management: in 68.2% of farms, the parking for visitors was either the same as the workers’ or not clearly marked. In 77.3% of farms, there were no dedicated locker rooms for visitors. However, all farms had a visitor logbook to register entries.

Regarding structural aspects, 27.3% of farms lacked an external fence to prevent the entry of wild animals and unauthorized visitors. In 21 out of 22 farms, no other livestock species were farmed. In over half of the farms, there were no measures in place to prevent bird access. All farms owned disinfectants with proven efficacy against swIAV.

Ventilation was also assessed under this criterion, with only one farm which had an air filtration system. The temperature was automatically managed in 77.7% of farms, but the humidity was recorded in less than 14%.

Finally, another critical item identified by experts was F2_4. However, the compliance rate for this item was 100%, as all farms declared having a procedure for cleaning and disinfecting facilities at the end of each production cycle.

### 3.3. Geographical Risk Assessment of Farms

Eight farms obtained the highest level of risk (5) for the location in a suitable area for H5Nx circulation in domestic species and eight for the location in a suitable area for H5Nx in wild species. Five farms received the highest score for being located in an area with both domestic and wild bird virus suitability. Only two farms received the lowest score (1) for wild birds’ virus suitability.

### 3.4. Farm Ranking

**Figure 3:**
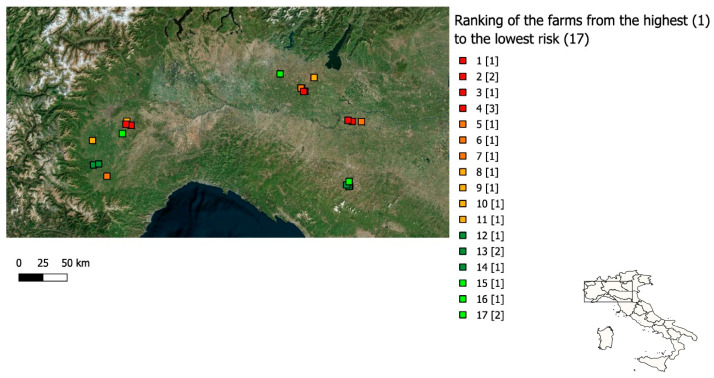
Ranking of the 22 assessed farms, from the one at the higher risk (1) to the one at the lower risk (17), obtained combining the six biosecurity RPCs and the geographical risk indicator.

Figure 3 shows the final ranking of the 22 assessed farms, obtained by combining the six biosecurity RPCs and the geographical risk indicator. A completed ranking was obtained, from the farm at the higher risk (1) to the one at the lower risk (17). Ties were observed in three pairs of farms and among three additional farms with identical scores, resulting in the same ranking.

## 4. Discussion

In this study, we performed a semi-quantitative risk assessment on swIA in pig farms, through the evaluation of biosecurity systems and the geographical location of farms. The FMEA method is a risk assessment tool that has recently been applied in the medical field [36,37,38]. A modified version developed by Franceschini and Galetto [31] has been adopted to evaluate the risk of African swine fever (ASF) introduction in both commercial [27] and outdoor pig farms [39]. We applied the method to a different hazard, for which specific biosecurity checklists and data are not available, combining the evaluation of biosecurity with expert opinion on SwIA. We relied on the opinion of experts in the sector of biosecurity and swine diseases to assign importance scores to the sub-criteria. Although this introduced some subjectivity in the assessment, it enabled us to supply to the scarcity of information related to specific biosecurity measures for swIA. According to this approach, it was possible to identify biosecurity measures considered as particularly relevant (critical items), whose non-compliance could compromise the whole biosecurity system. Therefore, higher risk was attributed to those farms with a high non-compliance score for important sub-criterions or non-compliance to biosecurity measures defined as ‘critically important’ by experts. We designed our checklist to evaluate different types of production systems, considering the specific characteristics and risk factors associated with each production type. Due to the limited number of assessed farms, which may not be fully representative of the Italian situation, it was not possible to identify a specific production type consistently at higher risk than the others. The ranking of farms in terms of risk should be referred to the population at hand, and generalization should handled with caution. However, the sample allowed highlighting clear differences in terms of risk among farms, thus providing a consistent ranking and proving the method to be effective. High animal density and a high number of farms are recognized risk factors for swine influenza in pigs [40,41,42]. For this reason, we selected our sample of farms in northern Italy, an area which accounts for most of the Italian pig population and has the highest animal density [26].

Close proximity to poultry is another acknowledged risk factor, since it increases the chance of interspecies transmission of IAV [12,43]. Thus, we used a geographical indicator of risk, related to suitable areas for H5Nx viruses in domestic and wild birds. The five farms scoring the highest RPC values were located in the province of Brescia; this province is bordering Verona province, which has the highest poultry farm density in Italy (0.36 poultry farms/km^2^, Italian average: 0.037 poultry farms per km^2^ [26]), and it is close to lakes hosting numerous aquatic bird species. Swine are susceptible to avian influenza virus, and the prevailing swine strains circulating in Europe derive from reassortments of avian and human viruses. Moreover, the infection of pigs with HPAI has been demonstrated both experimentally [44] and naturally [45]. When infected with other strains, swine may exhibit no clinical signs [45] or mild disease [44], with the likely consequence of undiagnosed virus circulation. This also happened in cattle infected by the zoonotic H5N1 virus in the USA, which likely derived from a single introduction from wild birds that possibly occurred months prior to its detection [46]. Therefore, biosecurity measures that prevent contact with birds must be included in the assessment, to account for the risk of introduction of avian strains that could remain undetected. In the evaluated farms, biosecurity measures related to bird control still represented a critical point, attributable to different factors. In particular, in 22.7% of farms, personnel were in contact with poultry, usually kept in small flocks close to the pig premises for household egg consumption. Moreover, although most of the farms adopted measures to limit contacts with wild mammals (e.g., wild boars), the possibility of interactions with wild birds remained relevant. This was mainly due to the absence of physical barriers, such as nets, which would prevent birds from accessing pig housing areas.

The risk of virus introduction through personnel was evaluated by criterion A, for which more than half of the farms showed medium risk (RPC = 3). Trainings on biosecurity, which was considered the most important sub-criterion overall by the experts (highest *I_b_*), had been recently carried out in 90% of the farms by all farmers. This showed a substantial improvement compared with results from Scollo et al. [27]; it is probable that the ASF epidemic, which has been ongoing in Italy since 2022, raised awareness among farmers on biosecurity. In addition, the Italian legislation issued with the Decree of 28 June 2022 (GU 173 of 26 July 2022) played a major role, as it made specific training on biosecurity mandatory for all workers in the swine sector. As previously mentioned, swine influenza is zoonotic, and humans can be infected through direct contact with infected pigs or contaminated environment [47]. Several studies have highlighted that occupational exposure to pigs increases the risk of swIAV in workers [48,49,50,51]. Although human seasonal influenza strains are different from those circulating in swine, health organizations recommend vaccination against seasonal flu for people in contact with animals that can harbor influenza virus [52,53]. Additionally, the use of protective equipment is strongly recommended to prevent zoonotic transmission [52,54]. In most of the farms in our study, personnel were not vaccinated, and the use of masks or goggles was very limited. The role of personnel is particularly relevant, especially considering that reverse zoonotic events are frequent, and infections of pigs with human strains are among the main cause of IAV diversity in pigs [8]. This could be explained by modern pig farming practices: large populations of piglets without a fully developed immune system are in contact with farmers, who have partial cross-immunity to influenza subtypes due to previous exposure to or vaccination against flu strains [8]. A study from Grøntvedt et al. [55], showed that the presence of workers with influenza-like illness was significantly associated with influenza in swine (OR = 4.15, CI 1.5–11.4, *p* = 0.005). Vaccinating workers should, therefore, be recommended also to limit the possibility of pigs becoming infected with human strains.

Criterion F, which relates to structures and access control, had the highest RPC (5) in all 22 farms, indicating the highest level of risk. This was mostly due to the lack of adequate visitor access control. The specific measure that triggered the highest risk score was the absence of restrictions for visitors with influenza-like symptoms, which were not implemented in any of the farms evaluated. On the contrary, measures to prevent unauthorized access of persons, animals, and vehicles, such as fences and gates, were implemented in the majority of farms, revealing again an improvement when compared with the results found by Scollo et al. [27]. Airborne transmission may play an important role in the dynamics of the disease in areas with a high density of farms. It is indeed common for farms in the same area to be infected at the same time, without animal movements being implied [23]. Parameters relevant for airborne transmission, such as temperature [24,56], can be controlled with forced ventilation systems. A study from Chantarizard et al. [57] showed higher thermal comfort in barns with mechanical ventilation, where animals were also less likely to be serologically positive for H1N1 compared with barns with natural ventilation. Among our farms, only one was equipped with an air filtration system, while the remaining units relied on natural ventilation or forced, but unfiltered, ventilation. Temperature and humidity were not always recorded.

Aspects related to management of premises were considered in criterion C, whose RPC distribution was not consistent among farms, probably due to the different risk factors and biosecurity measures adopted in the different production types evaluated. A complete all-in–all-out management for the whole farm or the application of a quarantine was considered a critical item (C2_1). Failure to adopt it in the fattening room is a risk factor for herds seropositivity [58]. In farrowing or farrow-to finish farms, where all-in–all-out operations are difficult, usually a quarantine for newly introduced animals is applied. The proper quarantine period to prevent the introduction of flu into herds was set to 6 weeks, which corresponds to the minimum period proposed in the Biocheck.UGent, for Porcine Reproductive Respiratory Syndrome virus. To our knowledge, no study has assessed the correct quarantine duration for flu. In our study, the length of the quarantine was not considered sufficient in half of the farms. In addition, no farms tested animals for flu before their introduction into farms. On the other hand, farms rearing sows were the only ones in our study to vaccinate animals against swine flu.

## 5. Conclusions

The proposed risk assessment method enabled highlighting differences among farms, identifying weaknesses that make some farms more at risk than the others. The assessment provided an overview of the biosecurity in a sample of farms. The sample selection, which was based on farmers’ willingness to participate, is a limit to our study. Although the sample may not be formally representative, the participating farms can be considered typical high-biosecurity farms of northern Italy. Consequently, the observed non-compliances with biosecurity are likely to represent an overestimation of the overall level of biosecurity in the area.

We propose the FMEA as a flexible and consolidated approach to assess the risk of introduction of different diseases in animal populations. If applied to a larger and more representative sample, the method could provide a broader risk ranking and could be used as a risk-based surveillance tool. This is particularly relevant for swIA in contexts where it is endemic, like Europe, and complete prevention of the virus introduction is challenging. While some measures, such as the use of personal protective equipment, are relatively easy to implement, others—like structural improvements to ventilation systems—require significant investment and are not always feasible.

The simultaneous circulation of different influenza strains within the same farm—and within the same animal—increases the chance of reassortment events. In this context, the biosecurity evaluation for swIA could integrate existing risk assessment frameworks (e.g., ClassyFarm in Italy) to assess the specific risk of pandemic influenza outbreaks. Risk-based monitoring strategies, together with recommendations for risk mitigation, could be focused on farms with a higher probability of viral introduction or persistence, improving early detection of reassortant strains—which may go undetected by passive surveillance systems relying only on clinical signs, enhancing the response and preparedness to the influenza threat.

## Figures and Tables

**Table 1:** Criteria in the swIA checklist used for data collection on farms.

Criterion	Brief Description
(A) Personnel	Measures concerning farm staff, such as entrance protocol, training received, and use of protective personal equipment
(B) Animal management	Measures related to animal health status, recording of production parameters, introduction frequency, and origin of incoming animals
(C) Shelter management	Quarantine procedures, management of sick pens, slurry handling, and cleaning and disinfection protocols
(D) Animal transport	Measures related to vehicles and areas dedicated to loading and unloading of animals
(E) Material management	Measures to control the entry and handling of feed, bedding, and enrichment materials
(F) Buildings and access control	Farm infrastructure-related measures such as perimeter barriers and gates, as well as visitor access and pest control strategies.

**Table 2:** Non-compliance scoring system for sub-criteria.

Non-Compliance Score for Sub-Criteria	Description
1	All items are satisfied
2	Between 62.6 and 99.9% of the items are satisfied
3	Between 37.6 and 62.5% of the items are satisfied
4	Between 0.1 and 37.5% of the items are satisfied
5	No items are satisfied, or at least one “critical item” is not satisfied

**Table 3:** Summary of importance scores for the sub-criteria (A1–F5).

	Sub-Criterion	Importance Score (5 = Highest Importance)
Criterion A	A1	4
	A2	3
	A3	4
	A4	5
Criterion B	B1	4
	B2	3
	B3	5
	B4	2
	B5	1
Criterion C	C1	4
	C2	5
	C3	3
	C4	1
	C5	2
Criterion D	D1	5
	D2	4
	D3	2
	D4	3
Criterion E	E1	3
	E2	5
	E3	4
	E4	2
Criterion F	F1	1
	F2	4
	F3	2
	F4	5
	F5	3

**Table 4:** Critical items, i.e., items selected as particularly relevant for swIA prevention and control by at least 13 out of 15 experts.

Item_ID	Item (Biosecurity Measure)	N° of Experts
A1_1	Change of clothes and footwear is carried out	13/15
C1_3	There is a physically separate dedicated premise for quarantine	13/15
C1_8	All-in/all-out and a suitable sanitary break period is practiced for the quarantine premises	13/15
C2_1	Management of all-in/all-out: it is complete, no temporal overlapping of batches in the whole farm, or quarantine is carried out	14/15
C2_5	A proper cleaning and disinfection protocol (including the correct cleaning and disinfection steps and the correct action times of the products used) is in place and written down	13/15
F2_4	There is a procedure/protocol of cleaning and disinfection of housing facilities and equipment after the end of each production cycle	13/15
F4_5	Visitors with clinical signs of influenza are not allowed to enter the farm	13/15

## Data Availability

The data presented in this study are available on request from the corresponding author due to privacy reasons.

## Supplementary Materials

The following supporting information can be downloaded at https://www.mdpi.com/article/10.3390/ani16040544/s1. Table S1: Checklist used for data collection containing 132 items A1_1-F5_6 for the evaluation of farm biosecurity. Table S2: Distribution of non-compliance scores obtained for the 27 sub criteria for the 22 evaluated farms.

## Abbreviations

The following abbreviations are used in this manuscript:

swIAV	Swine Influenza Virus
swIA	Swine Influenza
FMEA	Failure Mode and Effect Analysis
RPC	Risk Priority Code
ASF	African Swine Fever
