# Supplementary material for: Swine Influenza Virus Introduction in Pig Farms: A Semi-Quantitative Risk Assessment in Northern Italy
Source: Animals (Basel). 2026 Feb 10;16(4):544. doi: 10.3390/ani16040544 (PMC12937228; doi:10.3390/ani16040544)
Supplement: Supplementary file 1 [file animals-16-00544-s001.zip › animals-4089019-supplementary.pdf]

Supplementary Materials

Table S1: Checklist used for data collection containing 132 items A1_1-F5_6 for the evaluation of farm biosecurity

Criterion	Sub-criterion	Item ID	Item description
		A1_1	Change of clothes and footwear is carried out
		A1_2	Clothing and footwear are cleaned and disinfected regularly or are disposable
		A1_3	Personnel shower before entering the farm
		A1_4	Personnel wear safety mask as a personal protective equipment while in the farm
		A1_5	Personnel wear protective googles while working in the farm
		A1_6	Personnel wash their hands before entering premises
	A1<br><br>Use of personal protective equipment and clothing	A1_7	Personnel use gloves when handling animals, and the gloves are changed for each group of animals
		A2_1	There is a locker room with toilets and cleaners and a clear separation between clean and dirty areas: there are no areas of overlapping dirty-clean transit
		A2_2	There is a Danish entry, i.e. a bench that totally separates the dirty and clean areas and that remembers the personnel the threshold
		A2_3	If the farm has more than one production phase, the personnel do not work in different production units or, if they do, they change their clothes and shoes and they wash their hands
	A2<br><br>Entrance of personnel into the farm	A2_4	Personnel are vaccinated against human seasonal influenza
		A3_1	Staff has no other pigs
		A3_2	Staff has no contact with other pig holdings
		A3_3	Personnel have no poultry and have no contact with poultry farms
		A3_4	Staff does not engage in wild boar hunting activities
		A3_5	If it comes into contact with animals from other farms or suids (wild boars), the staff should rest for at least 48 h before entering the farm after this activity.
	A3<br><br>Contact of personnel with other pigs and poultry	A3_6	If personnel are exposed to pigs that test positive for infectious diseases, they avoid contact with other pigs on the farm (e.g. personnel exposed to sick pigs in one shed do not work in other sheds on the farm).
		A4.1a	All personnel working on the farm (workers, technicians, managers, keepers/owners) are adequately trained on biosecurity and the course is less than a year old
A	A4<br><br>Biosecurity training	A4.1b	All personnel working on the farm (workers, technicians, managers, keepers/owners) are adequately trained on biosecurity and the course is more than a year old

		A4.1c	Only part of the personnel working on the farm has received biosecurity training and the training is less than one year old.
		A4.1d	Only part of the personnel working on the farm is trained in biosecurity and the training is more than one year old.
		A4.1e	The farmer and staff are either not trained at all on biosecurity or there is no clear evidence of courses
B	B1<br><br>Animals health status/ identification/recording of movements and productions	B1_1	The health status of all animals for SwIAV is known, prior to their entry into the herd
		B1_2	Vaccination against the three main circulating subtypes of SwIAV (H1N1,H1N2,H3N2) is carried out
		B1_3	All animals are correctly identified on the farm
		B1_4	There is an accurate and punctual registration of movements in/out the holding structures
		B1_5	Any results of official or self-inspection analyses are present (samples taken from animals or other matrices)
		B1_6	There is a documented vaccination plan
		B1_7	There is a system for recording company health, breeding, reproduction, production data
	B2<br><br>Number of farms of origin of the introduced pigs	B2_1	Daily inspection of animals in pens/enclosures is carried out to check their health status.
		B2_2	The farmer has appointed a farm veterinarian
		B2_3	Management practices that encourage contact between animals (e.g., group mixing, or cross-fostering more than once) are not in place.
		B2_4	Animal handling equipment (e.g. pig snare) is not moved between production units or it is cleaned and disinfected before being used in another unit.
		B2_5	Family slaughter, if it takes place, takes place under veterinary supervision (Documentation is available)
		B2_6	There is a hygienic and sanitary procedure/protocol of handling equipment used for vaccine prophylaxis and therapeutic treatments
	B3<br><br>Number of farms of origin of the introduced pigs	B3_1a	The farm introduces animals throughout the year from one or two farms or does not introduce animals (e.g. farrow-to finish farm)
		B3_1b	The farm introduces animals throughout the year from three farms
		B3_1c	The farm introduces animals throughout the year from four farms
		B3_1d	The farm introduces animals throughout the year from five farms
		B3_1e	The farm introduces animals throughout the year from more than five farms
	B4<br><br>Number of animal introductions per year	B4_1a	The farm does not introduce animals through the year
		B4_1b	The farm introduces animals 1-2 times a year
		B4_1c	The farm introduces animals 3-5 times a year
		B4_1d	The farm introduces animals 6-12 times a year

		B4_1e	The farm introduces animals more than 12 times a year
	B5<br>Management of animals with an impaired growth	B5_1a	There are no "scraps" (animals with impaired growth) they are all euthanized, and the vehicle collects the scraps from a single collection point
		B5_1b	Scraps are taken away at the end of the cycle and the vehicle collects them from a single collection point
		B5_1c	Scraps are taken away at the end of the cycle and the vehicle does not collect it from a single collection point
		B5_1d	Scraps are taken away during the cycle and the vehicle collects them from a single collection point
		B5_1e	Scraps are taken away during the cycle and the vehicle does not collect them from a single collection point
C	C1<br>Quarantine	C1_1	A quarantine of at least 6 weeks is carried out (with animals serologically tested for diseases and visited)
		C1_2	If quarantine is used, animals in quarantine are inspected at least once a day so that any clinical signs can be detected at an early stage
		C1_3	There is a physically separate dedicated premise for quarantine
		C1_4	Quarantine ventilation systems are separate from the rest of the premises or the ventilation flow is from the housing premises to the quarantine
		C1_5	Biosecurity measures for staff access to the quarantine area are implemented
		C1_6	Quarantine premises have separate entrances
		C1_7	Quarantine premises have separate pits
		C1_8	All-in/all-out and a suitable sanitary break period is practiced for the quarantine premises
		C1_9	For quarantine management, staff change clothes entirely and use dedicated equipment
		C1_10	There is a protocol in place for the management of animals that have tested positive for a disease while in quarantine.
	C2<br>Internal animal flow and cleaning and disinfection procedures	C2_1	Management of all-in/all-out: it is complete, no temporal overlapping of batches in the whole farm or quarantine is carried out
		C2_2	At least 5 days of sanitary break between emptying and introducing the new animals is followed
		C2_3	Structures are meticulously washed using disinfectants between batches
		C2_4	Pressure equipment is available for cleaning, washing and disinfection
		C2_5	A cleaning and disinfection protocol is in place and written down
	C3	C3_1	The farm has physical or natural barriers that circumscribe the animal housing and management area

	Structure and building related to animal shelters	C3_2	The area of housing and management of animals has a wall or a fence suitable to prevent the entry of other animals including wild ones
		C3_3	There is a planimetry, with sheds and stalls univocally numbered, through which it is possible to verify the flow of the movements of the animals in the farm and identify the groups of animals.
		C3_4	Animals do not have the ability to access/live in the outdoor environment, or animals do have the ability to access an external area in which contact with other pigs, wild boars, other animals included birds and carcasses is prevented.
		C3_5	Livestock housing shall have airtight, well-maintained, seamless walls, floors, and windows and doors that can be effectively cleaned and disinfected
		C3_6	The structure of the buildings and the organisation of tasks allow a unidirectional flow of workers from units/sectors/pens with healthy animals to those with sick animals.
		C3_7	The structure of the buildings and the organisation of tasks allow a unidirectional flow of workers from the units/sections/pens with young animals to those with older animals.
		C3_8	The structure of the buildings and the organisation of tasks allow a unidirectional flow of workers from the units/sections/pens of the housing premises to the quarantine premises
	C4 Dead pigs management	C4_1	Where piglet carcasses are temporarily stored on the farm premises, pending their removal, the containers used shall be properly sealed and suitable for storage
		C4_2	Dead animal carcasses shall be removed from farm premises within 24 hours of death.
		C4_3	Dead animal carcasses shall be stored in a suitable, functioning insulated container or in a sealed cold storage room for disposal in accordance with health regulations
		C4_4	The area underneath the carcass container/cold storage room, is suitable for the collection of any leaking materials
		C4_5	The area underneath of the carcass container/cold storage room, is suitable for cleaning and disinfection
	C5 Sick pen	C5_1	There is a space (premise/pen) designated as a sick pen (it shall be different from the one designated for quarantine)
		C5_2	The sick pen is physically separated from the other pens (e.g. seamless walls, different building), and there is no possibility of direct contact between healthy animals and animals in the infirmary.
		C5_3	The sick pen has a ventilation system that is separate from the other pens/housing premises, or is arranged in such a way that the air flows from the healthy animals to the sick animals, or there is no exchange of air flows.
		C5_4	The sick pen has separated pits
		C5_5	Animals showing clinical signs (anorexia, coughing, fever, lethargy,…) are moved to the sick pen

		C5_6	Instruments/materials used in the sick pen are washed and disinfected regularly
		C5_7	Instruments used in sick pen are not used in the other pens/premises (e.g. pig snare, syringes)
D	D1 Live animal transport vehicles	D1_1	There is an area for the disinfection of live animal transport vehicles located near the access to the farm but separate from the area designated for housing.
		D1_2	No live animal transport vehicles enter the farm's clean area
		D1_3	All farm vehicles do not have access to other pig farms
		D1_4	Vehicles are cleaned and disinfected (including wheels and fenders) with disinfectants proven effective for SwIAV before entering the clean area
	D2 Animal unloading/loading	D2_1	There is a loading bay for loading and unloading animals
		D2_2	Specific gates are in place to prevent animals from turning back
		D2_3	The unloading/loading of live animals takes place outside the farm's clean area
		D2_4	The unloading/loading of live animals takes place with a single-load vehicle.
	D3 Carcasses disposal	D3_1	The carcass container/cold storage rooms are outside the clean area of the farm
		D3_2	Emptying the carcass container/storage room takes place by non-contact tipping directly into the carcass removal truck
		D3_3	The contents of the carcass container/cold storage rooms are transported by staff to the outside of the farm
		D3_4	The carcass removal truck does not enter the farm
	D4 Equipment and tools for loading/unloading live animals	D4_1	While loading animals, transporters help inside the truck but never enter any clean area of the farm, which is clearly demarcated
		D4_2	Clothing provided to transporters is company issued or freshly laundered
		D4_3	The loading bay and the tools used are never switched from the dirty to the clean area and vice versa
		D4_4	The loading bay and the tools used are disinfected after each use
		D4_5	The weighbridge for the live animal vehicles, if any, is located outside the animal housing and governing area
E	E1 Procedures for loading/unloading of feed and materials	E1_1	The unloading of feed, bedding, outdoor material, etc. takes place outside the perimeter of the clean area.
		E1_2	Vehicles and outside operators do not stop or pass through a clean area to reach silos/barn/depot
		E1_3	If for the retrieval of the materials to be brought in, company operators must step on a common unloading area with external operators or pass through a dirty area, they use dedicated clothing and footwear

		E1_4	The truck route is such that it crosses the operators' routes, but the operators adopt procedures to remedy the problem or the truck route does not cross the operators' routes
		E1_5	The silos and the hayloft are inside the farm perimeter but not in contact with animals and/or operator routes or are outside the farm perimeter
	E2<br><br>Feed and materials storage	E2_1	The external material (bedding, enriching material...) is stored for 30 days or treated with UV rays
		E2_2	Fresh grass and cereals are not fed to pigs unless they have been treated to inactivate the influenza virus or stored (in closed and covered rooms) for at least 30 days prior to feeding.
		E2_3	The material is stored in closed and covered premises
		E2_4	Feed storage is protected from animals by sparrow nets/sealing/covering
		E2_5	There is no exchange of materials, equipment and/or feed with other farms.
		E2_6	Materials (e.g. enriching material) and equipment that come into contact with pigs are regularly cleaned and disinfected or replaced
	E3<br><br>Slurry management	E3_1	The slurry tank is located outside the perimeter of the clean area
		E3_2	The slurry tank can only be reached by a route outside the perimeter of the clean area of the farm
		E3_3	The slurry tank is not accessible to wild animals
		E3_4	There are entrances for slurry transport operations differentiated from those of the animal housing /management area
		E3_5	Slurry of other origin is not spread in the fields adjacent to the farm
	E4<br><br>Vehicles for loading/unloading feed and materials	E4_1	Vehicles other than those for loading/unloading live animals and carcasses (transport of feed, materials, owned vehicles) do not enter the farm's clean area.
		E4_2	The vehicles are disinfected in advance on a dedicated yard and there is documentation
		E4_3	Vehicles neither transit nor are parked anywhere on the farm
		E4_4	Vehicles do not come into contact with the area where animals are present (clean area)
F	F1<br><br>Farm periemeter barrier	F1_1	There is an external fence for the entire farm perimeter that prevents the entrance of wild animals and visitors
		F1_2	Access points are closed by a gate
		F1_3	Signs prohibiting access for unauthorized persons are present and clearly visible at the entrance
		F1_4	The farm has a dedicated area, located before the entrance barrier, for the parking of staff vehicles.
		F1_5	The pavement around the farm is asphalted

		F1_6	There is no unused or waste material around the farm, the farm is well maintained, there are no bushes, brushwood around the farm
	F2<br><br>Other animals and general cleaning and disinfection procedures	F2_1	No pets are present and have access outside the farm perimeter
		F2_2	No other livestock species are farmed
		F2_3	Disinfectants with proven efficacy against swine influenza virus are available
		F2_4	There is a procedure/protocol of cleaning and disinfection of housing facilities and equipment after the end of each production cycle.
	F3<br><br>Pest and rodent control	F3_1	Rodent and pest control are checked at least every <6 months and there is documentation
		F3_2	Rodent control is carried out by an external specialized company
		F3_3	Pest control is carried out by an external specialized company
		F3_4	Sparrow nets are in placed or it is otherwise ensured that birds cannot enter the premises
	F4<br><br>Visitors	F4_1	The farm has a designated area, located before the entrance barrier, for visitors' vehicles to park
		F4_2	The farm has a dedicated filter area used as an hygiene lock for visitors
		F4_3	Rooms used as visitors' hygiene lock are adequately equipped with toilets and cleaners
		F4_4	There is an up-to-date visitors' logbook indicating at least the date, first and last name of the visitor, reason for the visit
		F4_5	Visitors with clinical signs of influenza are not allowed to enter the farm
		F4_6	Visitors wear masks when entering the housing facilities
	F5<br><br>Ventilation System	F5_1	There is an automatic temperature management system
		F5_2	There is an automatic system for recording the humidity.
		F5_3	There are air filtration and disinfection systems
		F5_4	Dust reduction systems are in place (e.g. barriers between farms or nebulisers on exhaust fans)
		F5_5	Ventilation is by forced air only
		F5_6	The air intakes are in a satisfactory state of cleanliness (e.g. the flaps in front of the fan lift)

Table S2: Distribution of non-compliance scores obtained for the 27 sub criteria for the 22 evaluated farms.

Criterion A

Sub criterion	Sub criterion non-compliance score	Number of farms	% of farms
A1 Use of personal protective equipment	1	0	0.00
	2	5	22.73
	3	12	54.55
	4	5	22.73
	5	0	0.00
A2 Entrance of personnel into the farm	1	3	13.64
	2	9	40.91
	3	4	18.18
	4	5	22.73
	5	1	4.55
A3 Contact of personnel with other pigs and poultry	1	0	0.00
	2	7	31.82
	3	15	68.18
	4	0	0.00
	5	0	0.00
A4 Biosecurity training	1	20	90.91
	2	2	9.09
	3	0	0.00
	4	0	0.00
	5	0	0.00
Criterion B			
Sub criterion	Sub criterion non-compliance score	Number of farms	% of farms
B1 Animals health status/ identification/recording of movements and productions	1	0	0.00
	2	21	95.45
	3	1	4.55
	4	0	0.00
	5	0	0.00
B2 Number of farms of origin of the introduced pigs	1	1	4.55
	2	16	72.73
	3	5	22.73

	4	0	0.00
	5	0	0.00
B3<br>Number of farms of origin of the introduced pigs	1	17	77.27
	2	1	4.55
	3	4	18.18
	4	0	0.00
	5	0	0.00
B4<br>Number of animal introductions per year	1	1	4.55
	2	8	36.36
	3	10	45.45
	4	2	9.09
	5	1	4.55
B5<br>Management of animals with an impaired growth	1	5	22.73
	2	16	72.73
	3	0	0.00
	4	1	4.55
	5	0	0.00
Criterion C			
Sub criterion	Sub criterion non-compliance score	Number of farms	% of farms
C1<br>Quarantine	1	2	25.00
	2	2	25.00
	3	0	0.00
	4	0	0.00
	5	4	50.00
C2<br>Internal animal flow and cleaning and disinfection procedures	1	12	54.55
	2	0	0.00
	3	0	0.00
	4	0	0.00
	5	10	45.45
C3<br>Structure and building related to animal shelters	1	3	13.64
	2	18	81.82
	3	1	4.55

	4	0	0.00
	5	0	0.00
C4<br>Dead pigs management	1	9	40.91
	2	10	45.45
	3	3	13.64
	4	0	0.00
	5	0	0.00
C5<br>Sick pen	1	2	9.09
	2	2	9.09
	3	15	68.18
	4	2	9.09
	5	1	4.55
Criterion D			
Sub criterion	Sub criterion non-compliance score	Number of farms	% of farms
D1<br>Live animal transport vehicles	1	0	0.00
	2	11	50.00
	3	5	22.73
	4	6	27.27
	5	0	0.00
D2<br>Animal unloading/loading	1	9	40.91
	2	8	36.36
	3	4	18.18
	4	1	4.55
	5	0	0.00
D3<br>Carcasses disposal	1	13	59.09
	2	6	27.27
	3	1	4.55
	4	1	4.55
	5	1	4.55
D4<br>Equipment and tools for loading/unloading live animals	1	11	50.00
	2	8	36.36
	3	3	13.64

	4	0	0.00
	5	0	0.00
Criterion E			
Sub criterion	Sub criterion non-compliance score	Number of farms	% of farms
E1 Procedures for loading/unloading of feed and materials	1	15	68.18
	2	0	0.00
	3	1	4.55
	4	1	4.55
	5	5	22.73
E2 Feed and materials storage	1	16	72.73
	2	6	27.27
	3	0	0.00
	4	0	0.00
	5	0	0.00
E3 Slurry management	1	2	9.09
	2	9	40.91
	3	8	36.36
	4	3	13.64
	5	0	0.00
E4 Vehicles for loading/unloading feed and materials	1	14	63.64
	2	5	22.73
	3	2	9.09
	4	0	0.00
	5	1	4.55
Criterion F			
Sub criterion	Sub criterion non - compliance score	Number of farms	% of farms
F1 Farm perimeter barrier	1	8	36.36
	2	9	40.91
	3	3	13.64
	4	2	9.09
	5	0	0.00

F2<br>Other animals and general cleaning and disinfection procedures	1	7	31.82
	2	15	68.18
	3	0	0.00
	4	0	0.00
	5	0	0.00
F3<br>Pest and rodent control	1	2	9.09
	2	6	27.27
	3	10	45.45
	4	4	18.18
	5	0	0.00
F4<br>Visitors	1	0	0.00
	2	0	0.00
	3	0	0.00
	4	0	0.00
	5	22	100.00
F5<br>Ventilation System	1	0	0.00
	2	3	13.64
	3	8	36.36
	4	6	27.27
	5	5	22.73
